# Supplementary material for: ECuADOR—Easy Curation of Angiosperm Duplicated Organellar Regions, a tool for cleaning and curating plastomes assembled from next generation sequencing pipelines
Source: PeerJ. 2020 Apr 7;8:e8699. doi: 10.7717/peerj.8699 (PMC7147433; doi:10.7717/peerj.8699)
Supplement: Supplemental Information 2 [file peerj-08-8699-s002.pdf]

Supplementary Table S2						
Organism	Introduced global variation	Ns in IR (Average)	Local variation detected in IR (Average)	Correct annotations	Incorrect annotation	length difference (in bp) when incorrect annotations (mean [min-max])
Arabidopsis thaniana	5E-05	9	0.0001	99.6	0.004	6 [2-8]
Arabidopsis thaniana	0.0001	16	0.0008	97.8	0.022	12 [2-15]
Arabidopsis thaniana	0.0003	28	0.0011	0.927	0.073	24 [2-68]
Arabidopsis thaniana	0.0006	55	0.0021	0.858	0.142	25 [2-64]
Arabidopsis thaniana	0.0008	84	0.0032	0.819	0.181	27 [2-133]
Arabidopsis thaniana	0.0015	139	0.0053	0.734	0.266	31 [2-168]
Arabidopsis thaniana	0.0017	168	0.0064	0.666	0.334	33 [2-173]
Arabidopsis thaniana	0.002	186	0.0071	0.624	0.376	38 [2-151]
Arabidopsis thaniana	0.003	278	0.0106	0.505	0.495	41 [2-207]
Arabidopsis thaniana	0.0078	735	0.028	0.154	0.846	97 [2-644]
Arabidopsis thaniana	0.015	1394	0.0531	0.028	0.972	370 [4-2159]
